# Empowering study of breast cancer data with application of artificial intelligence technology: promises, challenges, and use cases

**DOI:** 10.1007/s10585-021-10125-8

**Published:** 2021-10-26

**Authors:** Maryam Panahiazar, Nolan Chen, Dmytro Lituiev, Dexter Hadley

**Affiliations:** 1 Department of Surgery, School of Medicine, University of California San Francisco, San Francisco, CA USA; 2 Department of Computer Science, University of California Berkeley, Berkeley, CA USA; 3 Department of Clinical Sciences, College of Medicine, University of Central Florida, Orlando, USA; 4 Bakar Computational Health Sciences Institute, School of Medicine, University of California San Francisco, San Francisco, CA USA

**Keywords:** Artificial intelligence, Breast cancer study, Database, Machine learning, Deep learning, Electronic medical record

## Abstract

In healthcare, artificial intelligence (AI) technologies have the potential to create significant value by improving time-sensitive outcomes while lowering error rates for each patient. Diagnostic images, clinical notes, and reports are increasingly generated and stored in electronic medical records. This heterogeneous data presenting us with challenges in data analytics and reusability that is by nature has high complexity, thereby necessitating novel ways to store, manage and process, and reuse big data. This presents an urgent need to develop new, scalable, and expandable AI infrastructure and analytical methods that can enable healthcare providers to access knowledge for individual patients, yielding better decisions and outcomes. In this review article, we briefly discuss the nature of data in breast cancer study and the role of AI for generating “smart data” which offer actionable information that supports the better decision for personalized medicine for individual patients. In our view, the biggest challenge is to create a system that makes data robust and smart for healthcare providers and patients that can lead to more effective clinical decision-making, improved health outcomes, and ultimately, managing the healthcare outcomes and costs. We highlight some of the challenges in using breast cancer data and propose the need for an AI-driven environment to address them. We illustrate our vision with practical use cases and discuss a path for empowering the study of breast cancer databases with the application of AI and future directions.

## Introduction

Women experience certain health care challenges and are more likely to be diagnosed with chronic diseases and conditions such as cancer. Breast cancer is a leading cause of cancer death among women in the USA [1]. As artificial intelligence (AI) techniques have transformed health care and helped in treating diseases, one main interest is the possibilities of applying AI to breast cancer research and study. Computer-aided detection (CAD) was among the first few methods developed to assist mammogram interpretation [2]. Early studies [3] showed that the CAD technique was enough for detecting cancer. For example, it showed that the radiologists had a false-negative rate of 21% and the CAD method helped to reduce this false-negative rate by 77%. The study with the assistance of a CAD system [4] used 12,860 screening mammograms; each mammogram was interpreted without the assistance of CAD, followed by a reevaluation of the areas that are marked by the CAD system. Using CAD, the recall rate was improved from 6.5 to 7.7% and a 19.5% increase in the number of cancers were detected. However, this technique has failed to improve diagnostic performance due to increasing the high false-positive rates over time. Mammographic screening significantly reduces breast cancer deaths in women for a while [5]. Interpretation of mammograms is still very difficult and extensive experience is needed for accurate interpretation.

Our objective is to explore AI technologies and big data analysis in the context of empowering the study of cancer databases in particular. We present some use cases that highlight the opportunities for how big data along with AI and data analysis can be applied to deliver better use of data for improving outcomes and managing healthcare. Since this is a review paper to discuss the AI infrastructure in cancer study, the data that has been used in the use cases are not available and accessible to the public in this manuscript (some data are available through the related published paper). Our purpose is to show the promises, challenges, and limitations of AI infrastructure based on related works, our previous and ongoing research. This review paper discusses the following:

1. The characteristics of cancer study and its impact on healthcare.
2. The role of AI technologies such as deep learning for empowering cancer study.
3. The use cases to illustrate some of the limitations, challenges, opportunities and potential solutions in cancer study.
4. Conclusion and future of cancer research.

## Characteristics of cancer study and the impact of that on healthcare

The overarching goal of cancer study is to create a framework that leverages patient EMRs (e.g. images, clinical notes, reports, and structured data) to translate large-scale routine clinical datasets into the important knowledge for decision making. There are different kind of tests to detect breast cancer [6] such as imaging (radiology) tests for cancer, CT scans, MRI, X-rays and other radiographic tests, nuclear medicine scans, ultrasound, and mammograms. 3-D Mammograms are raising the bar on cancer detection rates and reducing false positives. For decades, mammograms have been the gold standard of breast cancer screening, but 3-D technology is helping improve the quality of breast imaging. 3-D mammograms (also called tomosynthesis) add another dimension to the traditional mammogram. Instead of just two views of the breast, 3-D mammograms allow radiologists to view from multiple angles. More images mean clearer detail, better detection, and fewer errors. Some studies have shown 3-D mammography may improve the detection of invasive breast cancers by 40% and reduce visits for follow-up tests by 15%. This technology is especially helpful for women with dense breast tissue, whose breasts are harder to examine with traditional mammograms, and who may be at higher risk of developing cancer. On average 30 millions of 3-D screening mammograms are performed annually. Based on American Cancer Society Guidelines for the Early Detection of Cancer in 2020, women age 45 to 54 years get mammograms every year.

Screening mammography has proven to be an effective tool for reducing breast cancer mortality by helping with the early detection of suspicious cases such as masses, architectural distortion, abnormal calcifications, and asymmetries. Sensitivity is reported upwards of 85%; however, this is accompanied by a high proportion of “recall” imaging for further evaluation of potentially suspicious findings. Per 1000 women who receive annual mammographic screening, approximately 80 are required to return for recall imaging, 30 must undergo biopsy, and this ultimately results in the detection of only eight cancers [7]. Therefore, there is significant room for improvement in reducing unnecessary recall imaging and biopsies. This could happen with extracting more information from images and/or other resources. There are several AI models for extracting information from recourses from traditional models to state-of-the-art AI platforms that we discuss in this article.

## The role of artificial intelligence (AI) technologies to empower cancer study

In 2015, deep learning significantly outperformed humans at image classification with more than 95% accuracy [8]. Better classifying of existing cancer can reduce overdiagnosis (decrease recall rate, decrease biopsy rate) and improve early detection with finding interval cancers. Deep learning models could be used in a wide variety of predictive problems and settings in health care. Deep learning models have also made big improvements to help identify, classify, and quantify patterns in medical images [9] specifically, due to the great improvements in computer vision, medical image analysis has gained improvements in image, computer-aided diagnosis, lesion detection, and microscopic imaging analysis [10].

Mammographic screening significantly reduces the breast cancer deaths in women. But, interpretation of mammograms is still very difficult [11] and extensive experience is needed for accurate interpretation. There has been new advancement in applying AI models to improve the false rates in mammographic interpretation where they can achieve similar performance to experts. In one of the recent study, they used large data sets and showed superior performance to radiologists in cancer detection. More specifically, they used a large data set from the UK (25,000 mammograms collected between 2012 and 2015) and a large enriched dataset from the USA (30,000 mammograms collected between 2001 and 2018). They used an ensemble of 3 deep neural networks where the inputs are mammogram images and the output is breast cancer risk score between 0 and 1. The results showed an absolute reduction for false positive of 5.7% in USA and 1.2% in UK and false negative of 9.4% in USA and 2.7% in UK [12]. These studies were a few examples to demonstrate how deep learning models have revolutionized women's health in breast cancer. In next section we discuss and review a few more previous and ongoing use cases from our research group.

## The Use cases to illustrate some of the limitations, challenges, opportunities and potential solutions in cancer study.

### Use case #1: large scale label extraction of pathology report to facilitate annotating the images for deep learning

**Fig. 1:**
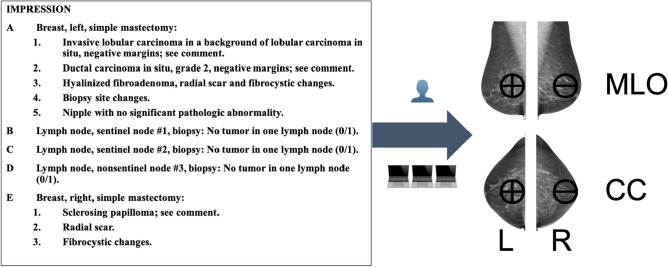
Example of pathology report in left side

As explained earlier, screening mammography is effective in reducing mortality but has a high rate of unnecessary recalls and biopsies. While deep learning can be applied to mammography, one of the challenges is the need for large-scale labeled (annotated) datasets, which are difficult to obtain. As part of our previous study [7], we demonstrate the feasibility of framework using machine learning models to derive pathologic diagnosis from free-text breast pathology reports. This annotation platform can be used to annotated the images for deep learning processing. About 7000 women (mean age = 51.8 years), ~ 10,000 (9787) reports were extracted from an in-house pathology database at UCSF. The “final diagnosis” section was considered in the analysis. All mammograms from 2000 to 2016 internal and external has been selected. We only included diagnostic screeners and MRI, and US are excluded. 3099 outcomes were manually labeled with expert as Bilateral(-), Left( +), Right( +), and Bilateral( +) [7]. An Example of breast pathology report is shown in Fig. 1.

**Fig. 2:**
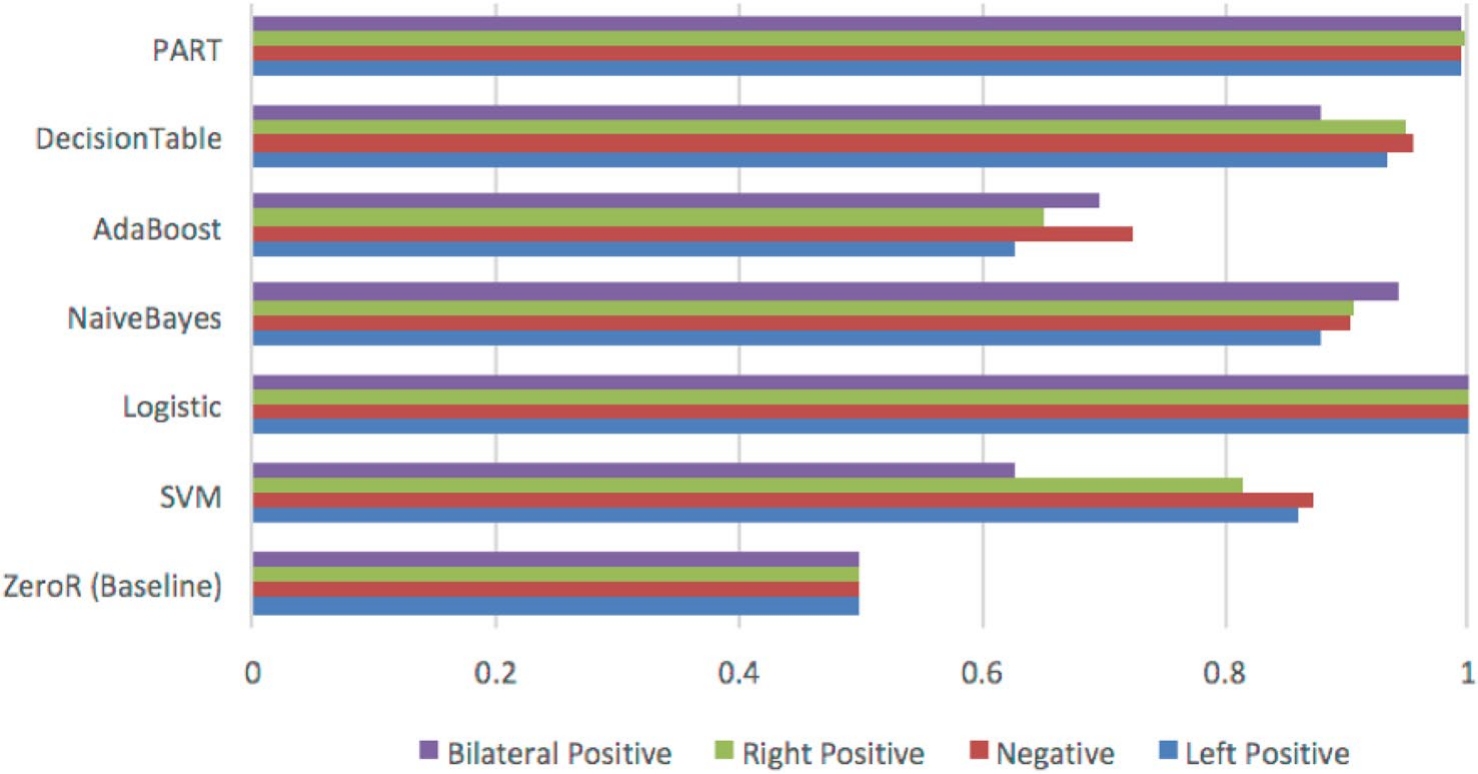
Performance (AUC) of six natural language classifier (NLC) models in comparison with baseline

The pathology report text was converted to the ARFF file format and imported to machine learning framework (e.g. Weka). Each report was first tokenized. In our case, each token was an N-gram (a set of co-occurring words) in lengths of one to three words. For example, the phrase “no ductal carcinoma” will result in the following tokens: “no,” “ductal,” “carcinoma,” “no ductal,” “ductal carcinoma,” and “no ductal carcinoma.” Each pathology report was tokenized into a vector of N-grams to serve as the input for TF-IDF (term frequency–inverse document frequency). Following the construction of the TF-IDF matrix, six supervised machine learning algorithms plus base line were tested to determine the best performing classifier for predicting the label for each report: PART, decision tables, AdaBoost, Naive Bayes, multiclass logistic regression, support vector machine (SVM), and majority vote classifier (ZeroR). For our dataset, logistic regression outperformed all other classifiers as shown in Fig. 2. AUC is calculated for each outcome (Right Positive = 0.989, Left Positive = 0.994, Negative = 0.987, Bilateral Positive = 0.983, and Weighted Avg. = 0.989). For overcome the challenges of preprocessing and coding pipeline, we demonstrated a hybrid framework using traditional models combined with IBM Watson to annotate over 10,000 breast pathology reports for the development of a large-scale database to be used for deep learning in mammography as reported by more detail in original manuscript [7].

### Use case #2: ontology development for smarter AI

**Fig. 3:**
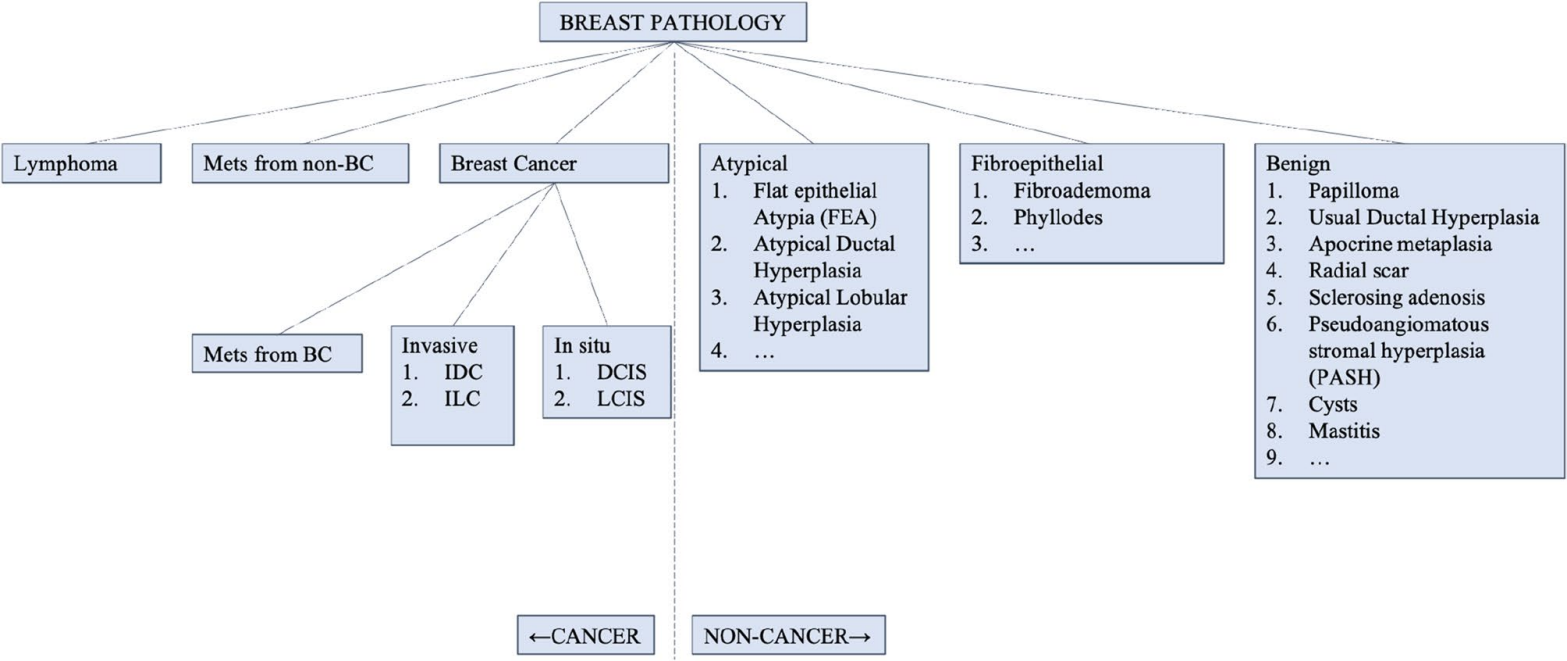
A foundation for ontology development for terms and concepts in pathology reports

Developing an ontology and using it to annotate the data and services in analysis workflows can provide a foundation for AI technologies, such as concept-based searches and comprehensive federated queries over data sources. There is as yet no computer-processable resource to describe all terms with the relations for pathology reports. Even though there are several ontologies and common data models such as SNOMED CT [13, 14] and cancer care treatment outcome ontology (CCTOO) which is about treatment end points in cancer, and to systematically capture and share outcomes data to inform better patient care. Campbell [15] developed the terminology defined by The CAP with SNOMED CT observable ontology. They defined 194 SNOMED CT observable entity concept definitions to represent required data elements for CAP colorectal and breast cancer synoptic worksheets. Even the terminologies that developed in this project established the model to characterize pathology data for information exchange, public health, and research analytics. But still it was a lack of fully defined the concepts in current pathology reports. To address these unmet needs (e.g. terms and concepts in pathology report, image related terms), we have built a foundation to develop the ontology (to be continued), to organize high-level and sub-branches of concepts with structured knowledge representation to facilitate standardized sharing of real-world data. An example of these concepts for cancer and non-cancer terms has shown in Fig. 3.

### Use case #3: using deep neural networks and “fastText” library

**Fig. 4:**
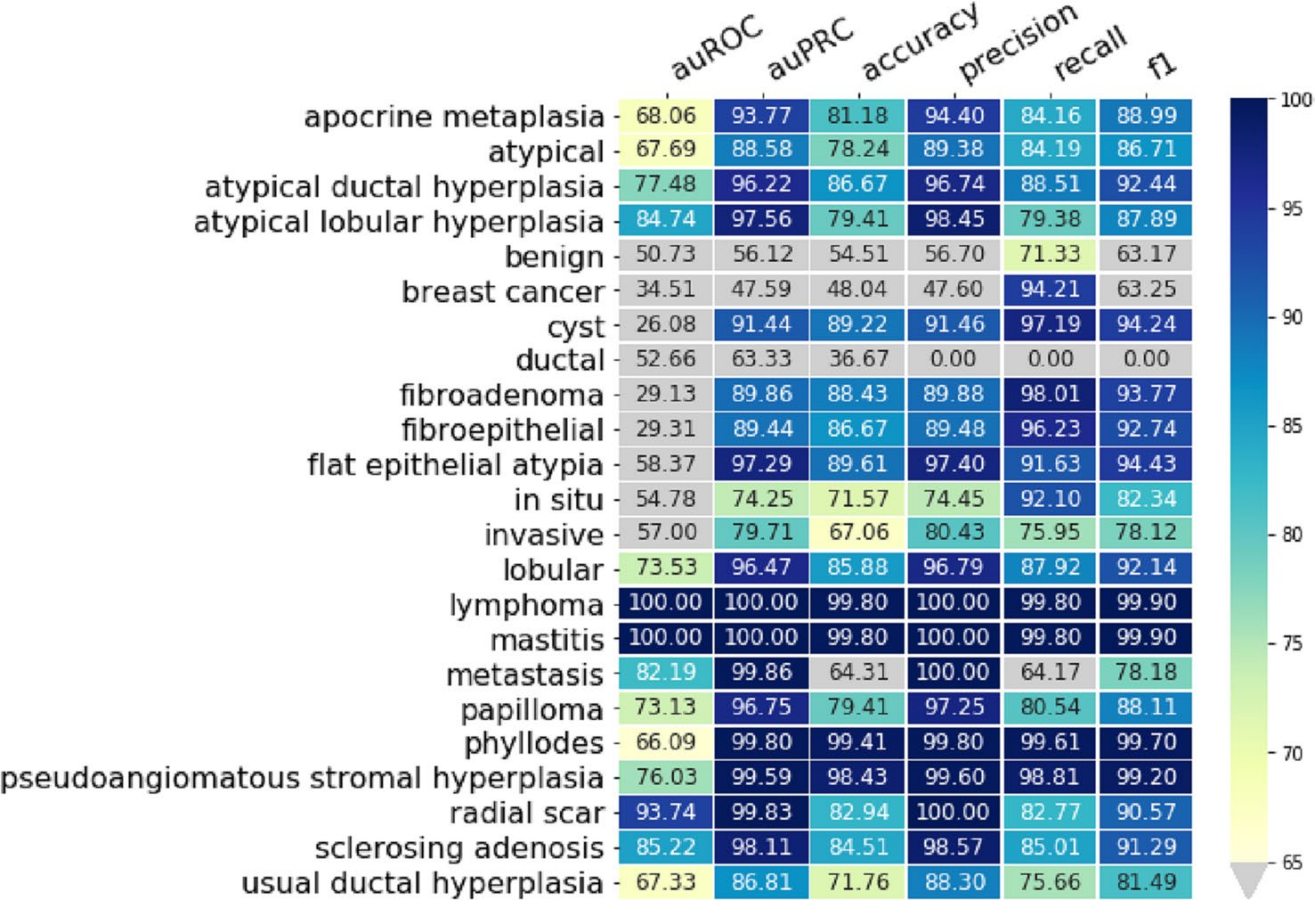
Performance table for fastText

Deep neural networks have recently become very popular for text processing. While these models achieve very good performance in limited laboratory practice, they can be slow to train and test, which limits their use on very large datasets and creates a big challenge. FastText [16] as a library for efficient text classification and representation learning helps solve this problem. It uses a hierarchical classifier instead of a flat structure, in which the different categories are organized in a tree (binary tree instead of list). This reduces the time complexities of training and testing text classifiers from linear to logarithmic with respect to the number of classes. In fastText, sentences are presented with bag of words and bag of n-gram. The information across classes are shared through the hidden representation. In one of our study for label extraction we used sate of the art fastText for label classifications. This use case in ongoing research and an example of performance table for fastText for several labels based on ontology from use case#2 is shown in Fig. 4. There are rooms to continue with other noble and state of the art platforms (e.g. Google ML, Microsoft Azure) for label extraction and classifications instead of traditional NLC models.

- Representing sentences with bag of words
- Representing sentences with bag of n-grams
- Using subword information
- Sharing information across classes through a hidden representation
- Using hierachical softmax that takes advantage of the unbalanced distribution of the classes to speed up computation
- Sharing information across classes through a hidden representation
- Using hierachical softmax that takes advantage of the unbalanced distribution of the classes to speed up computation

## Conclusion and future work

There are several AI Techniques that could be empower cancer study in different level of complexity as we discussed with their own challenges, promises and limitations. We are planning to expanding our research studies to process other medical records such as radiology reports and clinical notes as well as testing other automated and semi-automated solutions to help experts in any level of programming to take advantage of AI models in their research. We hope to design an automated pipeline for large-scale clinical data annotation so that existing clinical records can be efficiently utilized for the development of deep learning algorithms. We also offering the semi-automatic (human in the loop) annotation platform to make labeled data available for AI models. We aim to develop a curation tool able to translate a clinical (breast imaging) report into a mask outlining a lesion on a radiogram. The demand for this automation stems from the fact that development of clinical grade models for breast cancer detection requires datasets with explicit localization of lesions. However, creation of large extensively annotated mammography datasets remains a significant challenge that highlights the significant need of research and investigation in using AI infrastructure in cancer study.

## Abbreviations

AI: Artificial intelligence
ML: Machine learning
EMR: Electronic medical record
